# Low-Loss Buried InGaAs/InP Integrated Waveguides in the Long-Wave Infrared

**DOI:** 10.1021/acsphotonics.3c01898

**Published:** 2024-05-28

**Authors:** Miguel Montesinos-Ballester, Elsa Jöchl, Victor Turpaud, Johannes Hillbrand, Mathieu Bertrand, Delphine Marris-Morini, Emilio Gini, Jérôme Faist

**Affiliations:** † Institute for Quantum Electronics, ETH Zürich, CH-8093 Zürich, Switzerland; ‡ Centre de Nanosciences et de Nanotechnologies (C2N), Université Paris-Saclay, CNRS, 9112 Palaiseau, France

**Keywords:** low losses, passive waveguides, buried waveguides, mid-infrared waveguides, photonic integrated circuits

## Abstract

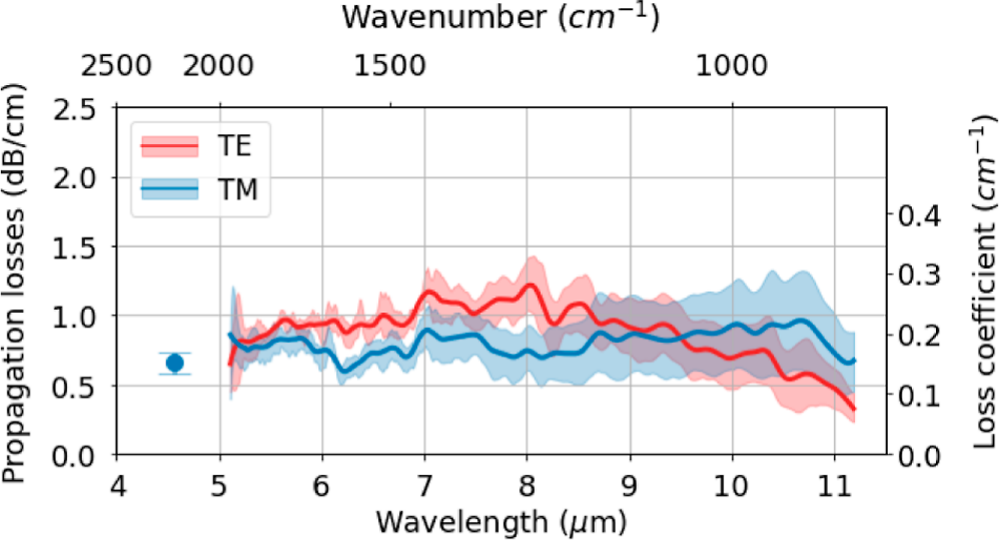

In this work, we present a photonic integrated platform based on buried InGaAs waveguides with InP cladding that operates over a large mid-infrared (mid-IR) spectral range. Thanks to wet-etch fabrication patterning and Fe doping, low propagation losses below 1.2 dB/cm (0.3 cm^–1^ loss coefficient) have been obtained between 4.6 and 11.2 μm wavelengths (890–1960 cm^–1^ wavenumber), in both transverse electric (TE) and transverse magnetic (TM) polarization modes. The possibility of monolithically integrating such waveguides with mid-IR sources offers promising perspectives for developing broadband, homogeneously integrated systems.

## Introduction

The mid-infrared (mid-IR) spectral range is typically defined from 2 to 20 μm wavelength (500–5000 cm^–1^ wavenumber) and has acquired significant relevance over the past two decades due to its vast number of applications, including free-space communications, thermal imaging, and sensing purposes. The long-wave infrared (LWIR) range is typically defined from 8 to 14 μm wavelength and is particularly interesting as many important molecules (such as ozone or alkanes) show their fundamental resonance in this range.^1^ In this regard, the development of broadband and low-loss photonic integrated waveguides operating in the LWIR is of great importance, as they can provide compact and robust devices.

To date, several mid-IR platforms have been demonstrated in different materials.^2,3^ However, mid-IR platforms are typically limited to wavelengths shorter than 5 μm as the LWIR presents different challenges. On one hand, most of the conventional materials used in photonic integrated circuits (Si, SiO_2_, SiN, SiC, sapphire, or LiNbO_3_) have a transparency window below 8 μm wavelength that prevents their use in the LWIR,^4,5^ and only recent works have employed Ge and SiGe-alloy materials^6−8^ to demonstrate losses below 4.6 dB/cm between 5 and 11 μm wavelengths.^9^ On the other hand, the free-carrier losses increase with the square of the wavelength.^10^ Therefore, high-quality materials are essential to achieving high-performance devices in the LWIR.

To operate in the LWIR, some works have reported the use of surface plasmon polariton waveguides, achieving propagation losses of 67.3 dB/cm at a 9.1 μm wavelength and providing monolithic integration with lasers and detectors based on III–V materials.^11^ Other works have employed hybrid integration of different materials, such as germanium on zinc selenide, demonstrating optical losses of 5.2 dB/cm at a 7.8 μm wavelength.^12^ Alternatively, III–V compounds are of high interest to develop high-performance mid-IR integrated waveguides, as they can have a wide transparency window and the current technology allows remarkably low free-carrier concentrations, in the order of or even below 10^15^ cm^–3^.^13^ Among them, InGaAs and InAlAs waveguides with InP cladding are particularly promising, given the technological maturity of current semiconductor quantum cascade lasers (QCL) based on that set of materials.^2,14,15^ For instance, InGaAs membrane waveguides have been reported in the literature, achieving 4.1 dB/cm propagation losses at a 6.1 μm wavelength.^16^ InGaAs/InP passive waveguides patterned by dry-etching have also been demonstrated, reporting ∼1.2 dB/cm propagation losses a near 5.2 μm wavelength^17^ and 2.9 dB/cm at a 7.4 μm wavelength.^18^ The use of proton implantation has also been reported as a method to reduce the free carriers of originally active waveguides of QCLs to make them passive, reporting losses of approximately 1.4 dB/cm at a 9.6 μm wavelength.^19^ Furthermore, the use of waveguides based on lattice-matched III–V materials enables the monolithic integration of QCLs, typically emitting in transverse magnetic (TM) polarization. For example, evanescent coupling between a QCL and an InGaAs/InP waveguide has been demonstrated in a homogeneous platform,^20−22^ and optical losses below 5 dB/cm were demonstrated in similar waveguides up to 11 μm wavelength in transverse electric (TE) polarization.^23^ Also, our group has recently demonstrated a butt-coupling configuration between a QCL and a passive InGaAs waveguide, both buried in InP.^24^ The propagation losses of the passive section were estimated to be 1.2 dB/cm near the 8 μm wavelength, deduced from the different laser performances.

In this work, we have studied and developed a photonic integrated platform based on buried InGaAs core waveguides with InP cladding that addresses the most important sources of propagation losses. Scattering losses are minimized by employing a wet-etch fabrication step and relatively large bending radii, and free-carrier absorption (FCA) has been reduced by Fe doping during material growth. Thanks to these approaches, we have experimentally demonstrated propagation losses below 1.2 dB/cm between 4.6 and 11 μm wavelength in both TE and TM polarization. Moreover, since the optical waveguides are completely buried in InP material, they can be butt-coupled to QCLs (and possibly detectors) with negligible coupling losses,^24^ and in this way, any deterioration of the waveguide sidewalls, such as oxidation or unwanted molecular contamination, is avoided. This latter aspect becomes critical due to the significant molecular absorption characteristics of the mid-IR.

## Methods

### Platform Materials

The materials chosen to develop high-performance integrated waveguides are InGaAs with InP cladding, as both are commonly used to fabricate QCLs.^14^ InGaAs is preferred to InAlAs, since it has a higher refractive index, with values of *n* ≈ 3.5 and *n* ≈ 3.2 in the mid-IR, respectively.^25,26^ The higher index contrast with the InP cladding (*n* ≈ 3.1) increases the optical confinement, which is beneficial to reduce the bending losses and allow more compact devices, among others. The ternary InGaAs compound is also chosen such that its crystallographic lattice is matched with the InP substrate (i.e., In_0.53_Ga_0.47_As), and thus, any defects due to strain are avoided.

Achieving low material doping levels is critical to minimize the propagation losses in the LWIR, as the FCA scales with the wavelength (λ) as *N* × λ,^2^ where *N* is the carrier concentration averaged over the waveguide mode.^10^ Therefore, to reduce the free-carrier concentration to the lowest possible level, both the InGaAs core and InP cladding materials are doped with Fe. As it is well known for the growth of semi-insulating InP, the Fe atoms create deep carrier donor states, pinning the Fermi level, and reducing the free-carrier concentration to negligible values.^27,28^

To study the benefit of an Fe-doped platform, three samples with a 2 μm thick InGaAs layer are grown on a high-resistivity InP substrate (Fe-doped semi-insulating substrate) by metalorganic vapor phase epitaxy (MOVPE). The Fe concentration of the three InGaAs layers is adjusted with the gas flux during the growth. Then, the overall background free-carrier concentration is experimentally characterized by Hall effect measurements, giving the following results. For no Fe incorporation, the Hall measurement indicates n-type background carriers with a concentration of 3.4 × 10^15^ cm^–3^. For moderate and strong Fe flux during growth, the concentration is considerably reduced to 2.8 × 10^15^ cm^–3^ and 1.2 × 10^15^ cm^–3^, respectively. However, full compensation was not achieved, even for the highest Fe flux during growth.

To better understand the origin of free carriers, capacitance–voltage (CV) profiling is performed along the InGaAs growth axis of the sample with no Fe incorporated. This characterization indicates a low n-type concentration of 1.3 × 10^15^ cm^–3^ in the InGaAs layer, but the value increases near the interface with the InP substrate, leading to the total averaged free-carrier concentration of 3.4 × 10^15^ cm^–3^ previously estimated by the Hall effect. This increase is related to the MOVPE growth, where precursor gases are flowing over the sample substrate for a short time at the beginning of the process, possibly leading to impurity incorporation in the interface between the InGaAs layer and the semi-insulating substrate. To minimize this undesired effect, a 1 μm thick InP layer can be grown before the InGaAs core layer, keeping this source of free carriers or impurities separated by 1 μm from the waveguide core, thus reducing its interaction with the optical mode. Similarly, another 1 μm thick InP layer is grown on top of the InGaAs layer in this MOVPE step to minimize this possible contamination in subsequent growth steps. However, the impurities caused by precursor gases in the last lateral growth of Fe-doped InP by MOVPE cannot be avoided. By doing so, the effective free-carrier concentration that interacts with optical mode can be certainly reduced to values below the ones previously estimated by Hall effect measurements (i.e., 2.8 × 10^15^ cm^–3^ in the case of moderately Fe-doped samples).

In the following samples, in combination with the addition of these top and bottom InP cladding layers, both InGaAs and InP materials are grown with moderate Fe doping. This Fe incorporation is expected to reduce the free-carrier concentration that interacts with the optical mode to an estimated value of 1 × 10^15^ cm^–3^. Considering the Drude model for InGaAs material with a relative effective mass of 0.043 and carrier lifetime of 0.15 ps, this n-type value will lead to reasonable propagation losses of 0.1–0.8 dB/cm (0.02–0.18 cm^–1^) at 5–11 μm wavelengths. These FCA values provide an estimation of the minimum propagation losses that can be expected in the waveguides. In fact, they are in good agreement with the losses later obtained experimentally, and in particular at the longest wavelengths, where the FCA is expected to be the main loss contribution.

### Waveguide Fabrication and Modeling

In addition to the intrinsic FCA of the material, we herein address two more main sources of propagation losses. The first loss mechanism is scattering from the waveguide sidewalls. The second is the absorption due to Ga or In oxides and organic molecules that could be present on the waveguide surface or sidewalls. To minimize their impact as well as provide fabrication compatibility with buried-heterostructure QCL fabrication processes, wet-etch patterning and buried passive waveguides are chosen. The fabrication process is as follows. First, a 1 μm thick InP/Fe layer is grown by MOVPE on a high-resistivity InP substrate, followed by a 2 μm thick InGaAs/Fe layer and another 1 μm thick top InP/Fe layer. Next, a SiO_2_ hard mask is patterned by laser lithography and reactive ion etching steps. Then, the waveguides are wet-etched with an isotropic HBr/Br/H_2_O solution (17:1:10 concentration in volume). Finally, the SiO_2_ hard mask is removed in hydrofluoric acid (HF) solution, and another 3 μm thick top and lateral InP/Fe cladding is grown by MOVPE. If a planarized surface profile is desired, an additional lateral InP/Fe cladding growth can be performed prior to the hard-mask removal. The different layers of InP/Fe and InGaAs/Fe of the sample used for optical measurement have been grown with a moderate Fe-doping concentration.

Although the cut-view profile of the waveguide can slightly differ depending on the crystallographic orientation, the relatively large bending radius of 600 μm makes an adiabatic transition between them. Therefore, as observed in other works, it does not induce large losses.^29^ Similarly, the wet-etch may produce slightly different waveguide widths along the sample due to liquid turbulences during the etching step. Also in this case, we can assume adiabatic transitions between the slightly different waveguide widths.

**Figure 1:**
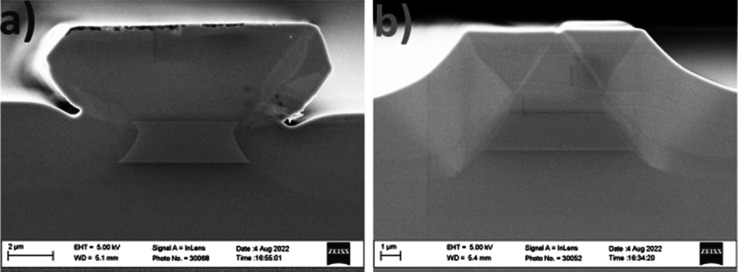
Scanning electron microscope images of the waveguide facet in the (a) [110] and (b) [−110] crystallographic orientation, which correspond to the *x*-axis and *y*-axis directions of the sample, respectively.

**Figure 2:**
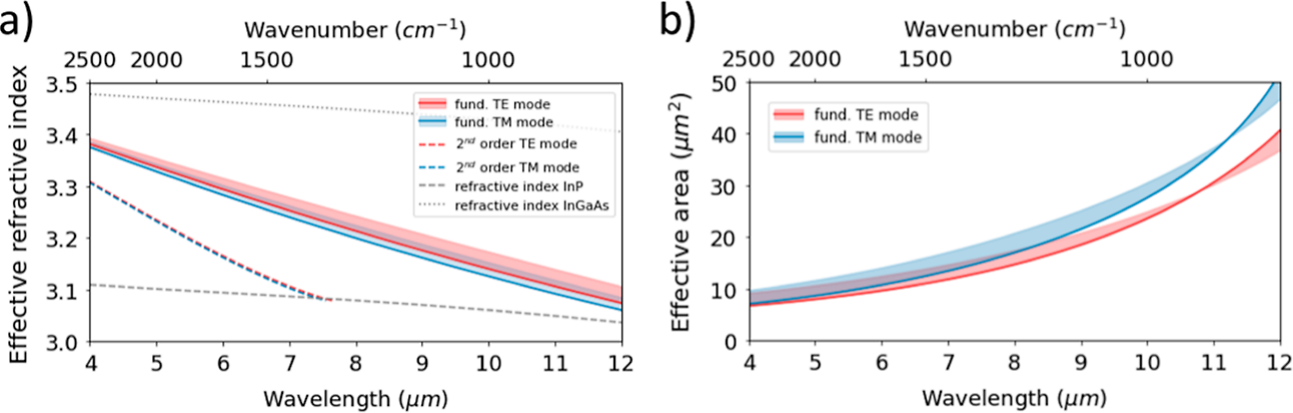
(a) Computed *n*_eff_ as a function of the wavelength or wavenumber of fundamental modes. The *n*_eff_ of the second-order modes of a 4.2 μm width waveguide is shown as colored dashed lines, which lie between the refractive index of bulk InGaAs and InP, shown as dotted and dashed gray lines, respectively. (b) *A*_eff_ as a function of the wavelength or wavenumber of the fundamental modes. In both graphs, the TE and TM polarization modes are shown in red and blue color, respectively. The values for the fundamental mode of a 4.2 μm width waveguide are depicted in solid, and the shading represents the range 4.2–6.2 μm width.

The fabricated waveguides have a width that can vary between 4.2 and 6.2 μm along the sample. These values were obtained by cleaving and taking several SEM images of the cross-section in different areas of the sample. As observed in Figure 2, numerical simulations indicate single-mode operation from 7.5 to 12 μm wavelength and in both TE and TM polarizations. However, when compared to air-cladded waveguides, the lower index contrast leads to larger effective areas (*A*_eff_), with values up to 40 μm^2^ at an 11 μm wavelength. Therefore, a relatively large bending radius must be employed (>400 μm) to achieve negligible bending losses. Numerical simulations show that the TE polarization mode exhibits slightly higher confinement than the TM mode for the entire spectrum under investigation. Furthermore, the effective refractive indices (*n*_eff_) of both fundamental modes are relatively close but do not cross each other or with second-order modes, which prevents mode crosstalk. As observed in Figure 1, the InGaAs waveguide core profile is not perfectly rectangular in the cleaved facets. Nevertheless, the trapezoidal cut-view profile of the fabricated waveguide core in both crystal orientations is expected to have a minor influence on the *n*_eff_ and modal area when compared to rectangular profiles.

### Waveguides Characterization

To characterize the propagation losses of this mid-IR platform, a set of 6 waveguides with increasing length from 1.9 to 9.9 cm is fabricated. To minimize the footprint of the device, we fabricated the waveguides in a spiral shape with a minimal bending radius of 600 μm. Since the waveguides are buried in InP material, which is transparent in the optical spectrum under test, the input and output facets are separated by 750 μm to avoid collecting any scattered light from the input facet.

**Figure 3:**
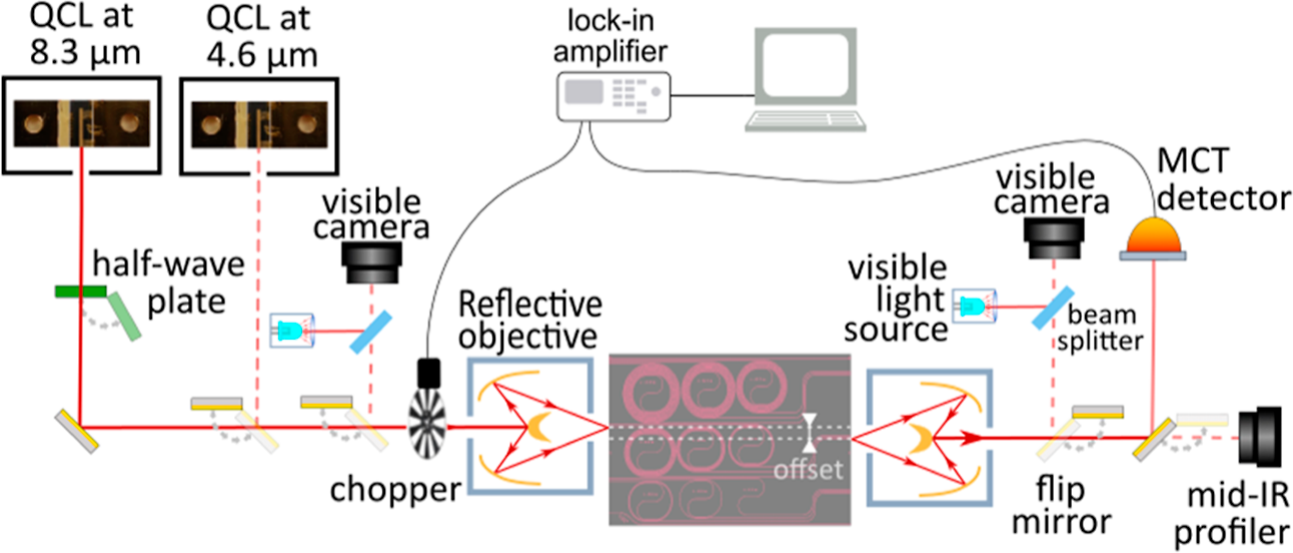
Schematic of the experimental setup used for propagation loss characterization.

To perform experimental measurements, the free-space characterization setup illustrated in Figure 3 was used. In that, two single-line, continuous-wave QCLs emitting near 4.6 and 8.3 μm wavelengths (2190 and 1200 cm^–1^ wavenumber) are installed. Since these QCLs emit in TM polarization, a half-wave plate is optionally used for the 8.3 μm emission QCL to rotate to TE polarization. To couple into the waveguides, a pair of reflective objectives and a system of visible cameras and flip mirrors are used. The output light is sent into a mercury cadmium telluride (MCT) detector and a bolometric mir-IR beam profiler is also used to ensure correct coupling to the waveguide.

## Results and Discussion

**Figure 4:**
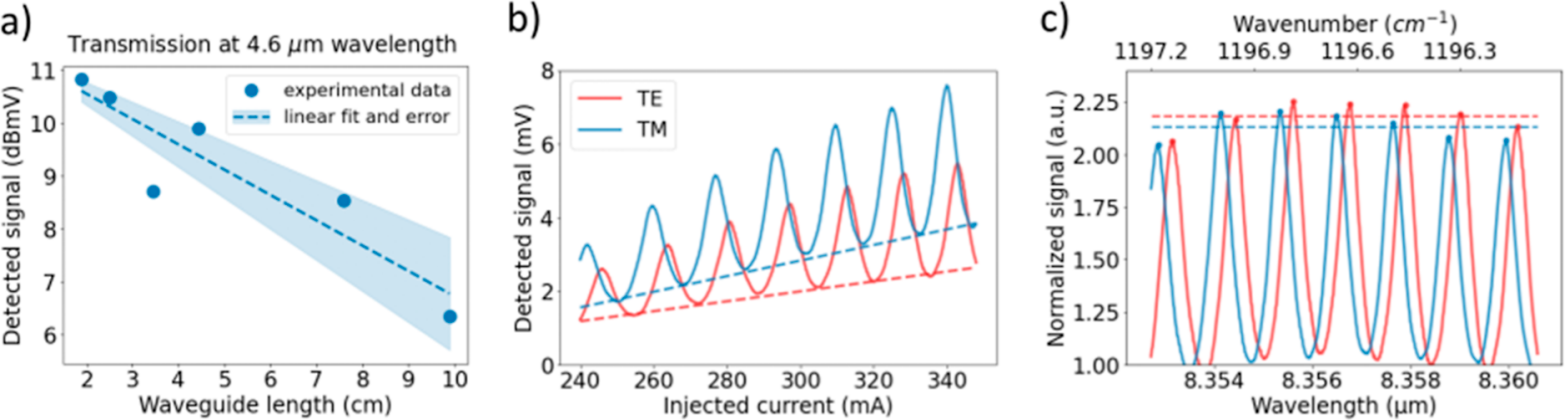
(a) Cut-back method measurements near a 4.6 μm wavelength. Dots: fitted transmission. Dashed line: linear fit of the data points. Shaded color: estimated linear fitting error. (b) Fabry–Perot method measurements near 8.3 μm wavelength. Dashed lines: bottom baseline used for normalization. (c) Normalized transmission spectrum near 8.3 μm wavelength. Dashed lines: average of fringe peaks used for loss calculations. For the three graphs, TE and TM polarizations are shown in red and blue colors, respectively.

Since the waveguides are multimode near the 4.6 μm wavelength, the propagation losses are first obtained at this wavelength by the non-destructive cut-back method in TM polarization. To avoid any undesired multimode behavior, the QCL emission is tuned by current, and the detected signal is smoothed with a Savitzky–Golay filter. This smoothed signal is then plotted in a logarithmic scale in Figure 4a, where the propagation losses are directly obtained from the linear regression of the data points, resulting in a propagation loss value of 0.5 ± 0.1 dB/cm (0.11 ± 0.005 cm^–1^). The linear fit value is obtained so that the sum of the squared residuals is minimized, and the error of the measurements is estimated from the standard deviation of this fitting parameter (linear slope) in the optimization algorithm (shown as the shaded color in Figure 4a). The experimental insertion loss is estimated to be 10.8 dB per facet at a 4.6 μm wavelength by measuring the optical power before injecting to the waveguide and calibrating the MCT detector.

The setup of Figure 3 is also used to characterize the propagation losses near 8.3 μm wavelength in TE and TM polarizations. Since the waveguides are single mode near that wavelength, and for simplicity, propagation losses are obtained in this case by using the interferometric spectral pattern of the Fabry–Perot cavity formed between the waveguide facets. To that end, the spectral emission of the single-line QCL is first characterized as a function of the injected current, so it is possible to finely tune it in a controlled manner. Then, the output signal is collected and plotted in Figure 4b. Since the QCL intensity increases when increasing the injected current, the signal is normalized by its linear baseline (dashed lines of Figure 4b). Finally, the propagation losses can be deduced from the interference fringes as in eq 1,^30,31^ where γ is the ratio between the maximum and minimum of the interferences (γ = *I*_max_/*I*_min_) that can be directly obtained from the peaks of Figure 4c (dash lines). In this characterization, a straight waveguide of 0.84 cm length (*L*) was used, and a facet reflectivity (*R*) of 0.27 was assumed from numerical simulations. The calculated propagation losses near the 8.3 μm wavelength are 1.75 and 1.91 dB/cm (0.404 and 0.439 cm^–1^) in TE and TM, respectively. The lower detected signal in TE polarization (compared to TM) is attributed to the losses introduced by the half-wave plate.
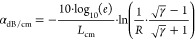
1

To obtain the propagation losses at longer and intermediate wavelengths, an alternative experimental setup that covers from 5.1 to 11.2 μm wavelength (890–1960 cm^–1^ wavenumber range) is used. This setup employs a commercial mid-IR source composed of four QCLs that can be continuously tuned by using an external cavity configuration. Since the sources are operating in a pulsed current injection regime, the line width of the source is relatively broad, and the cut-back method is preferred for propagation loss measurements, rather than via Fabry–Perot interferences. The optical transmission spectrum is obtained by collecting the light in a broadband MCT detector and tuning the mid-IR source in steps of 0.01 μm wavelength. To avoid any possible misalignment of the four QCLs that compose the mid-IR source, the coupling has been optimized for each waveguide with increasing length so that a maximum transmission is achieved at the central emission wavelength of each QCL: 5.6, 6.4, 7.8, and 9.6 μm wavelength. A bolometric mid-IR beam profiler is similarly used to ensure an appropriate coupling. To perform the measurements in both TE and TM polarization, we used a free-space polarization rotator too.

The experimental transmission spectrum of each waveguide is numerically smoothed with a Savitzky–Golay filter (3rd order and wavelength window span of 0.2 μm) to neglect the multiple atmospheric absorption peaks. Then, linear fit and error calculations are performed for each wavelength data point, similarly to the previous cut-back method measurements near a 4.6 μm wavelength. The obtained loss values are between 0.5 and 1.2 dB/cm (0.1–0.3 cm^–1^) in the entire characterized spectral range (5.1–11.2 μm wavelength or 890–1960 cm^–1^ wavenumber).

**Figure 5:**
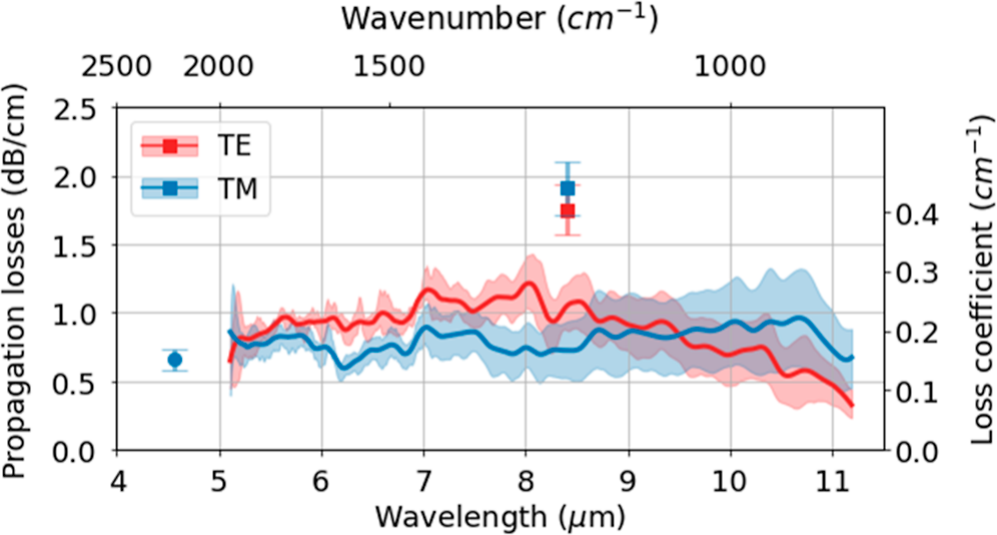
Summary of the experimental results of the propagation losses in TE (red) and TM (blue) polarizations of the moderate Fe-doped waveguides. Squared marker: results obtained by Fabry–Perot interferometry, where the error is estimated from the standard deviation of the fringe peaks. Round marker: cut-back method results. Solid line: cut-back method results. Shaded color: estimated error from the linear fit.

The different experimental results are summarized in Figure 5. As observed, losses below 2 dB/cm are obtained for both polarizations and the entire spectral range under consideration. A difference of nearly 1 dB/cm is found near 8.3 μm wavelength between Fabry–Perot and cut-back methods. The higher losses in the Fabry–Perot method can be explained by a combination of different factors: (i) residual polarization mode crosstalk produced in the two facet reflections and bends due to the nonsquared waveguide cross-section profile, (ii) an overestimated value of the facet reflectivity (*R* = 0.27 considered in measurements) due to misalignment during the lithography pattern, leading to a nonideal but uniform angle between the waveguide and the cleaved facet (assumed to be 90°), (iii) bandwidth and stability of the QCL emission, and (iv) collected scattered light from the input facet due to a relatively short (0.84 cm) and straight waveguide, leading to an intensity increase of the fringes’ minimal value. Hence, the results performed by the cut-back method are considered more accurate.

The relative flatness of the losses over the mid-IR spectrum is attributed to a balance of the different loss sources. In the lower wavelength range, the main contribution is attributed to scattering due to sidewall roughness and lithography imperfections, while a combination of free-carrier and material absorption is considered predominant in the LWIR. These results are also in good agreement with the value of 1.2 dB/cm near 8 μm wavelength estimated in previous works based on similarly buried InGaAs/InP waveguides.^24^ The TE mode shows marginally higher optical losses than TM at the lower and intermediate characterized wavelengths. This is attributed to scattering from the sidewalls of the waveguide, whose polygonal cross-section profile changes sequentially as it alternates between orthogonal crystallographic orientations in the bends. These scattering losses are expected to affect the TE mode more than the TM mode due to its electric field orientation.^32^ At the longer characterized wavelengths, these scattering losses have less impact, and reduced propagation losses are observed in TE due to its superior optical confinement, which minimizes the impact of impurities and free carriers at the cladding interfaces, as previously discussed.

## Conclusions

In this work, we have studied and addressed the main sources of propagation losses in the mid-IR to develop an integrated platform that achieves loss values of 0.5–1.2 dB/cm (0.1–0.3 cm^–1^) from 4.6 to 11.2 μm wavelengths (890–2190 cm^–1^ wavenumber) and also identified the potential impact of precursor contaminations during the MOVPE growth as a possible route for improvement. Interestingly, the possibility of monolithically integrating such passive waveguides with mid-R sources and detectors is of great interest. Hence, this work paves the way toward the development of fully integrated and high-performance systems operating over a broad bandwidth of the mid-IR spectrum. This realization could have a major impact in many areas, such as high-sensitivity and multimolecule sensors used in applications as diverse as environmental monitoring, hazard detection, industrial process control, or astronomy.

## Supporting Information Available

Schematic of the fabrication process flow of the passive waveguides, where the free carriers (impurities) caused by the precursor gas flow at the beginning of a MOVPE growth steps are clearly indicated; and further details on the theoretical values of insertion losses and experimental measurements of propagation losses in the alternative experimental setup from 5.1 to 11.2 μm wavelengths (PDF)
