# Supplementary material for: Low-Loss Buried InGaAs/InP Integrated Waveguides in the Long-Wave Infrared
Source: ACS Photonics. 2024 May 28;11(6):2236–41. doi: 10.1021/acsphotonics.3c01898 (PMC11192022; doi:10.1021/acsphotonics.3c01898)
Supplement: Supplementary file 1 — ph3c01898_si_001.pdf [file ph3c01898_si_001.pdf]

Low-loss buried InGaAs/InP integrated waveguides in the long-wave infrared

Miguel Montesinos-Ballester[1,*], Elsa Jöchl[1], Victor Turpaud[2], Johannes Hillbrand[1], Mathieu Bertrand[1], Delphine Marris-Morini[2], Emilio Gini[1], and Jérôme Faist[1]

[1] Institute for Quantum Electronics, ETH Zürich, CH-8093 Zürich, Switzerland

[2] Centre de Nanosciences et de Nanotechnologies (C2N), Université Paris-Saclay, CNRS, 91120 Palaiseau, France

*Correspondence: mmontesinos@ethz.ch

Supporting information

A theoretical insertion loss value of 7 dB per facet is obtained in the experimental setup of Figure 3 of the manuscript, which comprises obscuration of the reflective objective, mode mismatch between the waveguide and the input beam, and facet reflectivity of the fundamental optical mode

To obtain the propagation losses at longer and intermediate wavelengths, an alternative experimental setup that covers from 5.1 to 11.2 μm wavelength (890-1960 cm-1 wavenumber range) is used. The experimental transmission spectrum of each waveguide is numerically smoothed with a Savitzky-Golay filter (3rd order and wavelength window span of 0.2 μm) to neglect the multiple atmospheric absorption peaks, clearly visible in the 5.5-7.0 μm wavelength

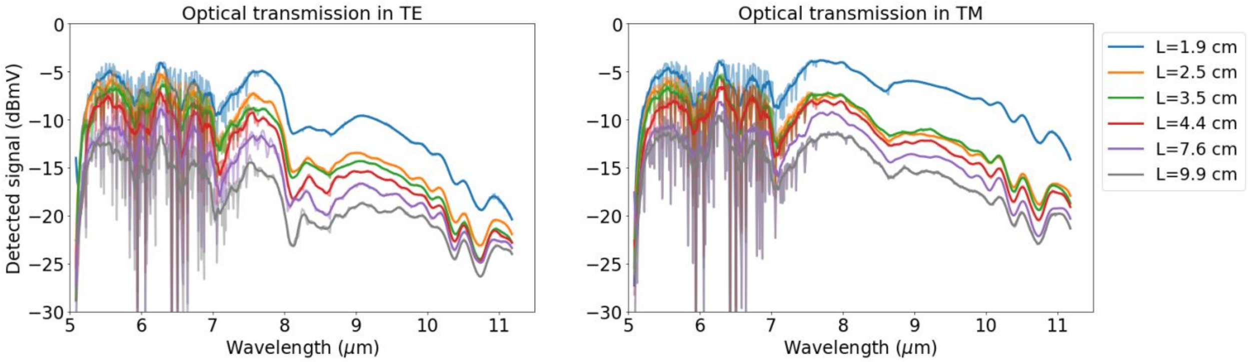

S1. Experimental transmission in TE (a) and TM (b) polarization for the different waveguides with increasing length from 1.9 to 14.2 cm. Shaded color: experimental data. Solid line: data smoothed with a Savitzky-Golay filter.

The next figure shows a schematic of the fabrication process flow of the integrated passive waveguides. First, a 1 µm thick InP layer is grown on top of a semi-insulating (Fe-doped) InP substrate, followed by a 2 µm thick InGaAs layer, and another 1 µm thick InP layer. These 3 layers are grown with moderate Fe-doping concentrations by adjusting the gas flow in the MOVPE process step. Then, a 400 nm thick SiO2 hard-mask is deposited by plasma-enhance chemical vapor deposition (PECVD) and a photoresist (PR) layer is spin-coated on top of it. The waveguides design is patterned in the PR by laser lithography, and transferred to the hard mask by reactive ion etching (RIE). After cleaning the PR, the waveguides are patterned by wet-etching in a HBr:Br:H2O (17:1:10 in volume) solution. The hard-mask is then removed in HF (1:5 in volume) solution. Finally, a 3 µm thick Fe-doped InP layer in grow by MOVPE.

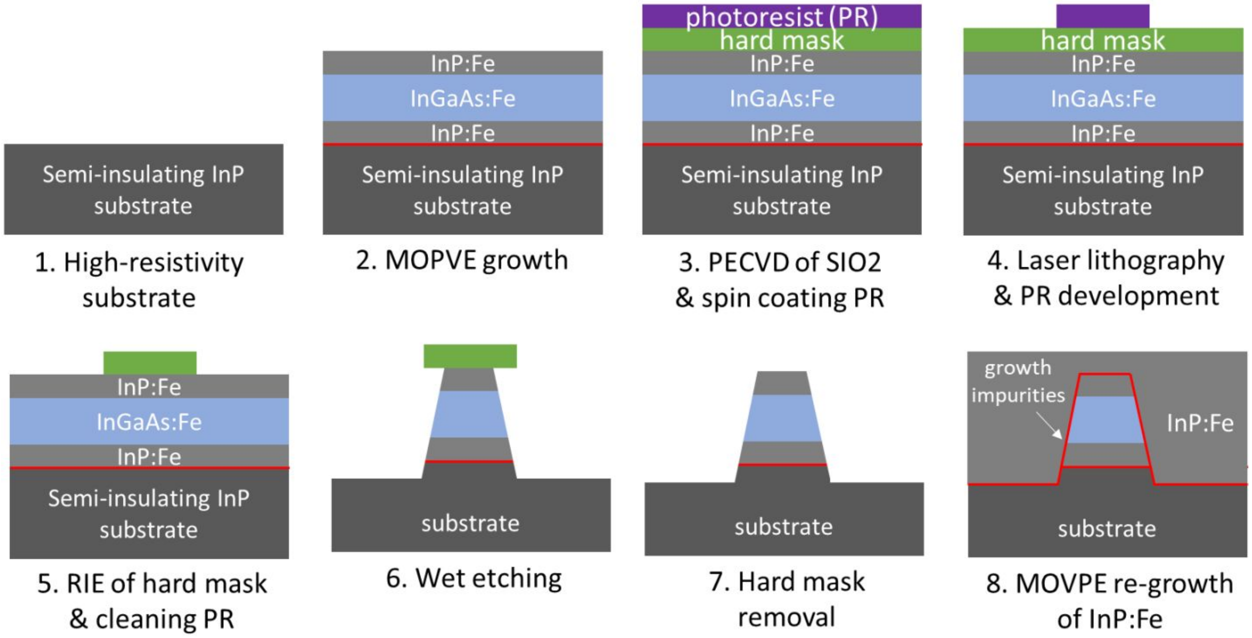

S2. Schematic of the passive waveguides fabrication process flow. The growth impurities caused by the precursor gas flow at the beginning of the MOPVE growth step are indicated in red color.
